# Association between cyclin D1 G870A polymorphism and cervical cancer risk: a cumulative meta-analysis involving 2,864 patients and 3,898 controls

**DOI:** 10.1186/s13000-014-0168-x

**Published:** 2014-09-10

**Authors:** Yuan-Yuan Hu, Rong Zheng, Chong Guo, Yu-Ming Niu

**Affiliations:** Department of Stomatology, Taihe Hospital, Hubei University of Medicine, 32 South Renmin Road, Shiyan, 442000 China; Department of gynaecology and obstetrics, Taihe Hospital, Hubei University of Medicine, 32 South Renmin Road, Shiyan, 442000 China; Clinical and Evidence-Based Medicine Center, Taihe Hospital, Hubei University of Medicine, Shiyan, P. R. China

**Keywords:** CCND1, Polymorphism, Cervical cancer, Meta-analysis

## Abstract

**Background:**

Association between Cyclin D1 (CCND1) polymorphism and cervical cancer risk are conflicting with published articles. We performed a meta-analysis to investigate the association between CCND1 G870A polymorphism and cervical cancer risk.

**Methods:**

PubMed, Embase and CNKI data were researched to conduct a meta-analysis on the associations between CCND1 G870A polymorphism and cervical cancer risk. Ten published case–control studies including 2,864 patients with cervical cancer and 3,898 controls were collected in this meta-analysis. Odds ratio (OR) with 95% confidence interval (CI) were applied to assess the relationship; meta-regression, sensitivity analysis and cumulative analysis were also conducted to guarantee the strength of results.

**Results:**

Overall, no significant association between CCND1 G870A polymorphism and cervical cancer risk were found in allele contrast (A vs. G: OR = 1.02, 95% CI = 0.88-1.19, *P* = 0.76 *I*^*2*^ = 74.5%), codominant model (GA vs. GG: OR = 0.98, 95% CI = 0.77-1.26, *P* = 0.90 *I*^*2*^ = 69.1%; AA *vs* GG: OR = 1.03, 95% CI = 0.75-1.41, *P* = 0.85 *I*^*2*^ = 75.9%), dominant model (GA + AA vs. GG: OR = 1.00, 95% CI = 0.78-1.28, *P* = 0.99 *I*^*2*^ = 72.3%) and recessive model (AA *vs* GG + GA: OR = 1.06, 95% CI = 0.85-1.23, *P* = 0.62, *I*^*2*^ = 70.1%). Similarly, in the stratified analysis by ethnicity, study design and genotyping type, no significant association detected in all genetic models either.

**Conclusions:**

Our meta-analysis indicated that CCND1 G870A might be not the crucial risk factor for the development of cervical cancer.

**Virtual Slides:**

The virtual slide(s) for this article can be found here: http://www.diagnosticpathology.diagnomx.eu/vs/13000_2014_168

## Background

Cervical cancer is one of the most common malignant diseases; it is the third most commonly diagnosed cancer and the fourth leading cause of cancer death in females with approximately 529,800 new cases and 275,100 deaths among females in 2008 worldwide [1].

Cervical cancer is a multifactorial and multistep disease. Innate immune deficiency, environmental aggravation, and genetic mutation have been considered as important pathopoiesis factors. New molecular epidemical studies revealed that human papillomavirus (HPV), particularly HPV 16 and 18 infections may be the common and important factor contributing to the development of cervical cancer, which is known to cause approximately 70% of cervical cancers [2,3].

Abnormal cell proliferation is an important step in cancer development. Cyclins are a family of proteins that control cell progression through the cell cycle by activating cyclin-dependent kinase (CDK) enzymes [4]. Cyclin D1 (CCND1) is a major regulatory protein that serves a critical function in the transition from G1 to S phase by binding to CDK4 and CDK6 to promote cell cycle progression during cell division [5]. Over-expression of CCND1 will induce tumor cells to pass the G1/S checkpoint of the cell cycle. Several studies have found that amplification of CCND1 and the aberrant expression of protein are associated with cell proliferation and poor prognosis in some cancers, such as head and neck cancer [6], lung cancer [7], and breast cancer [8].

Recently, molecular epidemiologic studies have directed considerable attention toward the association between genetic mutation and cancer susceptibility. Single-nucleotide polymorphisms (SNPs) are the most common type of genetic variation among people. The change in a nucleotide may alter gene functions and may influence protein expression, which could inhibit or promote cell proliferation and increase susceptibility to cancer development. In 1995, Betticher et al. [9] reported a synonymous SNP (G870A) in the CCND1 gene. The A allele has a longer half-life than the G allele and has been postulated to increase CCND1 level. Such increase promotes the proliferation of abnormal cells and the escape of these cells from apoptosis.

Catarino et al. published the first research about the association between CCND1 G870A polymorphism and cervical cancer risk in 2005 [10]. Since then, a large number of epidemiological studies have been conducted, but conclusions were inconsistent. In 2014, a recent meta-analysis was conducted by Wu et al., which only demonstrated that no significant association existed between CCND1 G870A polymorphism and cervical cancer risk. More detailed analysis on subgroup of study design, genotyping type and heterogeneity was not conducted [11]. We therefore performed the comprehensive meta-analysis to clarify the relationship between the CCND1 G870A polymorphism and cervical cancer risk with all published studies.

## Methods

### Search strategy

Three electronic databases, including Pubmed, Embase, and China National Knowledge Infrastructure (CNKI), were searched with the terms “CCND1”, “Cyclin D1”, “cervical cancer”, and “polymorphism” for studies published from 1995 to June 2014. Additional studies were identified through manual searches of the references of original studies or review articles on the topic of interest. Only studies published in English or Chinese were included. All studies selected for our meta-analysis met the following criteria: (a) observational (case–control or prospective) studies on the association between CCND1 G870A polymorphism and cervical cancer risk, (b) sufficient published data for estimating an odds ratio (OR) and 95% confidence interval (CI), and (c) the largest or most recent samples were selected when they overlapped with other studies.

### Data extraction

Data from all included studies were extracted independently by two investigators (Niu and Zheng). The extracted data included the name of the first author, publication date, country of origin, sources of controls, racial descent of the study population (categorized as Asian, Caucasian, and Mixed), number of different genotypes, and Hardy-Weinberg equilibrium (HWE) in controls.

### Statistical analysis

The strength of the association between CCND1 G870A polymorphism and cervical cancer was evaluated by ORs with 95% CI, comprised with allele contrast (A vs. G), codominant model (GA vs. GG, AA vs. GG), dominant model (GA + AA vs. GG), and recessive model (AA vs. GG + GA). The HWE of the control group was assessed, and a *P* value of less than 0.05 was considered significant disequilibrium. Stratified analyses were used to evaluate ethnicity, study design, and genotyping type technique. Heterogeneity was explored with the use of a chi-squared test, and the quantity of heterogeneity was measured by the *I*^*2*^ statistic. *I*^*2*^ values of 25%, 50%, and 75% represent low, moderate, and high heterogeneity, respectively. The OR of each model was estimated by using the fixed-effects model (Mantel–Haenszel method) when *I*^*2*^ ≤ 50%; otherwise, the random-effects model (DerSimonian and Laird method) was used. Meta-regression analyses were performed to assess potential covariates that can predict intertribal heterogeneity. Publication bias was assessed on the basis of modified Egger’s bias test and Begg’s funnel plot. Statistical analysis was performed by using STATA versions 11.0 (Stata Corporation, College Station, TX). Two-sided *P* value (*P* < 0.05) was considered statistically significant.

## Results

### Study characteristic

**Figure 1:**
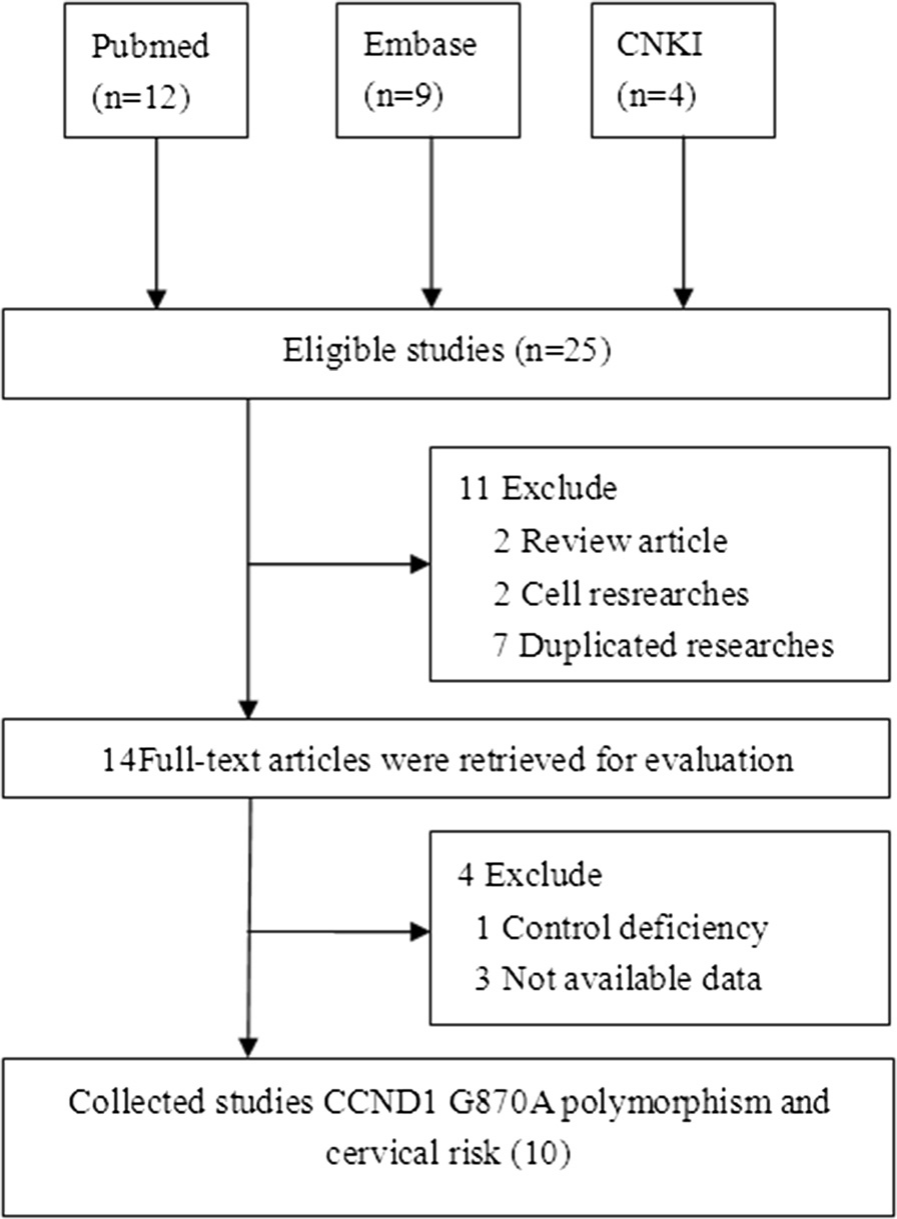
**Flow diagram of the study selection process.**

A total of 25 possible articles were searched in Pubmed (*n* = 12), Embase (*n* = 9), and CNKI (*n* = 4) (Figure 1). Nine duplicates and three irrelevant references were excluded through abstract screening. Ten published case–control studies involving 2,864 patients with cervical cancer and 3,898 controls met our inclusion criteria [10,12-20]. The data included from each study on different populations are presented in Table 1. Only two studies deviated from HWE in control populations [13,15].

**Table 1:** **Characteristics of case–control studies on CCND1 G870A polymorphisms and cervical cancer risk included in the meta-analysis**

**First author**	**Year**	**Country**	**Racial/descent**	**Source of controls**	**Case**	**Control**	**Genotype distribution**						***P*** **for HWE** ^**a**^	**Genotying type**
							**Case**			**Control**				
							***GG***	***GA***	***AA***	***GG***	***GA***	***AA***		
Catarino	2005	Portugal	Caucasian	Healthy base	143	103	35	64	44	9	55	39	0.091	PCR-RFLP
Jeon	2005	Korean	Asian	Hospital base	222	314	49	112	61	80	160	74	0.730	PCR-RFLP
Catarino	2008	Portugal	Caucasian	Hospital base	226	247	60	103	63	40	138	69	0.037	PCR-RFLP
Satinder	2008	India	Asian	Healthy base	150	150	33	64	53	30	65	55	0.184	PCR-RFLP
Thakur	2009	India	Asian	Hospital base	200	200	39	94	67	47	119	34	0.006	PCR-RFLP
Castro	2009	Sweden	Caucasian	Population base	952	1713	229	463	260	465	837	411	0.367	Multiplex PCR and hybridization
Ni	2011	China	Asian	Hospital base	300	312	48	160	92	70	137	105	0.051	PCR-RFLP
Warchoł	2011	Poland	Caucasian	Healthy base	129	288	35	65	29	116	123	49	0.100	PCR-RFLP
Wang	2012	China	Asian	Population base	327	411	86	180	61	92	203	116	0.859	PCR-RFLP
Djansugurova	2013	Kazakhstan	Caucasian	Healthy base	215	160	54	103	58	41	78	41	0.752	Direct sequencing

^a^HWE in control.

### Meta-analysis

**Figure 2:**
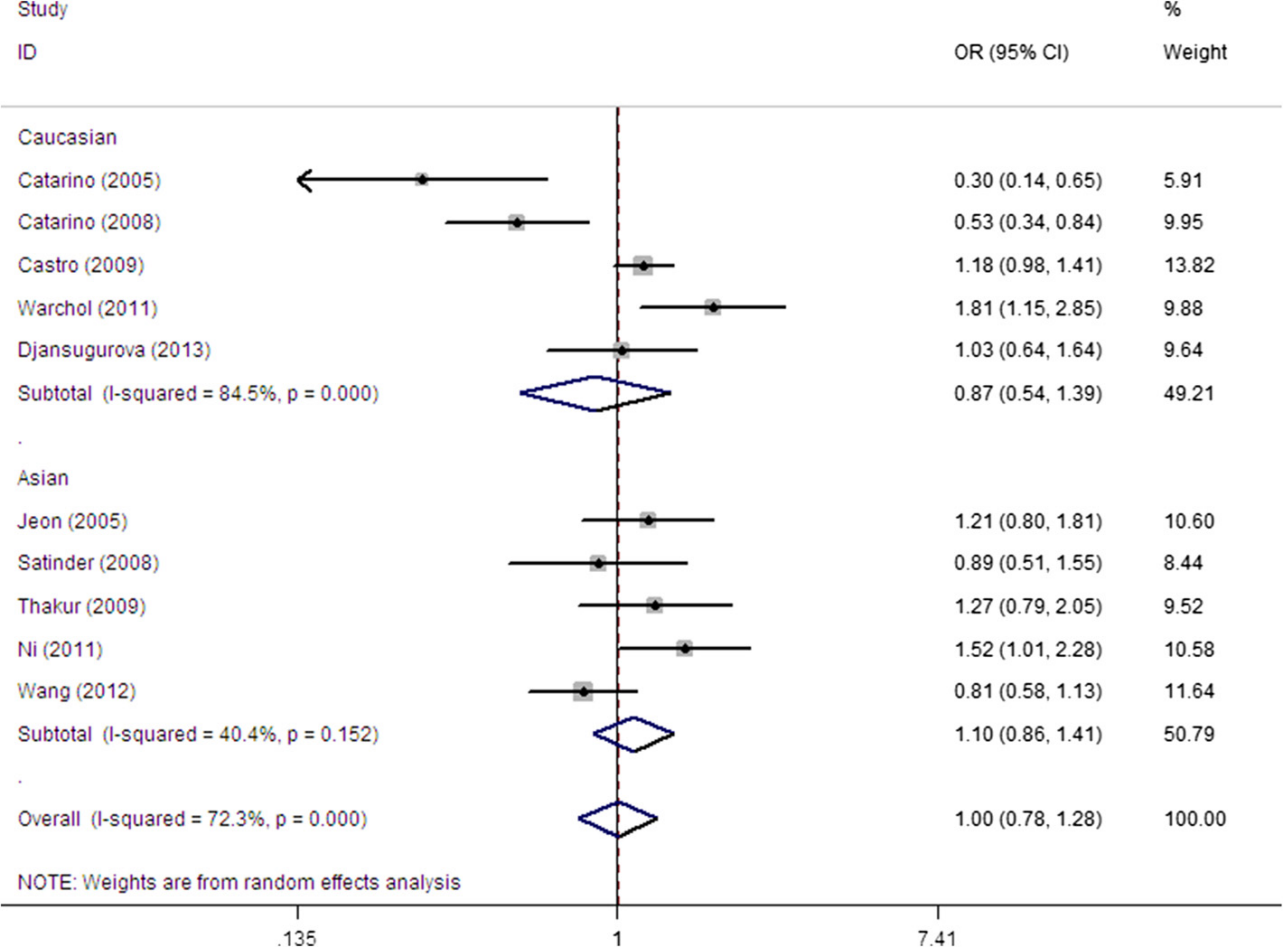
**OR of cervical cancer associated with CCND1 G870A polymorphism for the GA + AA vs. GG model in total.**

Overall, no significant association was found between CCND1 G870A polymorphism and cervical cancer risk in this meta-analysis (Table 2). Values of ORs with 95% CI were as follows: allele contrast (A vs. G: OR = 1.02, 95% CI = 0.88-1.19, *P* = 0.76 *I*^*2*^ = 74.5%); codominant model (GA vs. GG: OR = 0.98, 95% CI = 0.77-1.26, *P* = 0.90 *I*^*2*^ = 69.1%; AA *vs* GG: OR = 1.03, 95% CI = 0.75-1.41, *P* = 0.85 *I*^*2*^ = 75.9%); dominant model (GA + AA vs. GG: OR = 1.00, 95% CI = 0.78-1.28, *P* = 0.99 *I*^*2*^ = 72.3%, Figure 2); and recessive model (AA *vs* GG + GA: OR = 1.06, 95% CI = 0.85-1.23, *P* = 0.62, *I*^*2*^ = 70.1%). In the succeeding analysis of HWE studies, similar associations were found. In the stratified analyses of ethnicity, study design, and genotype, no significant association was found between CCND1 G870A polymorphism and cervical risk in almost all models. Heterogeneity was observed in all five genotype models. Meta-regression and stratified analyses were conducted, but no critical factors were found to explain heterogeneity in the subgroup of ethnicity, design, and genotype either (e.g., GA + AA vs. GG model: *P* = 0.321 for ethnicity, *P* = 0.819 for design, and *P* = 0.398 for genotype).

**Table 2:** **Summary ORs and 95% CI of CCND1 G870A polymorphisms and cervical cancer risk**

		**A vs. G**				**GA vs. GG**				**AA vs. GG**				**GA + AA vs. GG**				**AA vs. GG + GA**			
	N*	**OR**	**95% CI**	***P***	**I** ^**2**^ **(%)** ^**a**^	**OR**	**95% CI**	***P***	**I** ^**2**^ **(%)** ^**a**^	**OR**	**95% CI**	***P***	**I** ^**2**^ **(%)** ^**a**^	**OR**	**95% CI**	***P***	**I** ^**2**^ **(%)** ^**a**^	**OR**	**95% CI**	***P***	**I** ^**2**^ **(%)** ^**a**^
Total	10	1.02	0.88-1.19	0.76	74.5	0.98	0.77-1.26	0.90	69.1	1.03	0.75-1.41	0.85	75.9	1.00	0.78-1.28	0.99	72.3	1.06	0.85-1.32	0.62	70.1
HWE	8	1.01	0.86-1.18	0.92	71.9	1.08	0.85-1.39	0.52	62.2	1.00	0.72-1.39	0.99	73.1	1.06	0.82-1.36	0.66	68.7	0.97	0.79-1.20	0.79	59.8
Ethnicity																					
Caucasian	5	0.99	0.79-1.25	0.95	78.1	0.84	0.52-1.35	0.47	82.8	0.92	0.56-1.52	0.75	79.8	0.87	0.54-1.39	0.55	84.5	1.13	0.97-1.30	0.11	1.3
Asian	5	1.05	0.84-1.33	0.65	76.1	1.11	0.88-1.40	0.38	25.6	1.13	0.70-1.83	0.61	77.1	1.10	0.86-1.41	0.44	40.4	1.06	0.68-1.65	0.79	83.9
Design																					
Healthy base	4	0.98	0.71-1.36	0.91	76.8	0.88	0.47-1.64	0.70	78.1	0.90	0.45-1.78	0.76	77.5	0.89	0.47-1.67	0.71	81.3	1.02	0.79-1.31	0.91	6.7
Hospital base	4	1.11	0.87-1.40	0.40	71.7	0.99	0.60-1.63	0.96	79.4	1.24	0.75-2.07	0.40	74.5	1.06	0.68-1.66	0.80	76.6	1.25	0.82-1.89	0.30	77.1
Population base	2	0.94	0.64-1.39	0.76	91.0	1.08	0.91-1.28	0.38	0.0	0.87	0.39-1.95	0.73	91.2	1.00	0.70-1.44	0.99	72.7	0.85	0.42-1.70	0.64	92.0
Genotyping type																					
PCR-RFLP	8	1.00	0.82-1.23	0.97	78.3	0.94	0.67-1.33	0.73	75.3	0.98	0.64-1.51	0.93	79.6	0.96	0.68-1.34	0.79	77.3	1.04	0.77-1.40	0.80	74.9
Other	2	**1.12**	**1.01-1.25**	**0.03**	**0.0**	1.11	0.92-1.33	0.27	0.0	**1.25**	**1.02-1.54**	**0.03**	**0.0**	1.16	0.97-1.37	0.10	0.0	1.17	0.99-1.39	0.06	0.0

^*^Numbers of comparisons ^a^Test for heterogeneity. The significance of the bold values are all 0.03.

### Sensitivity analysis and cumulative analysis

**Figure 3:**
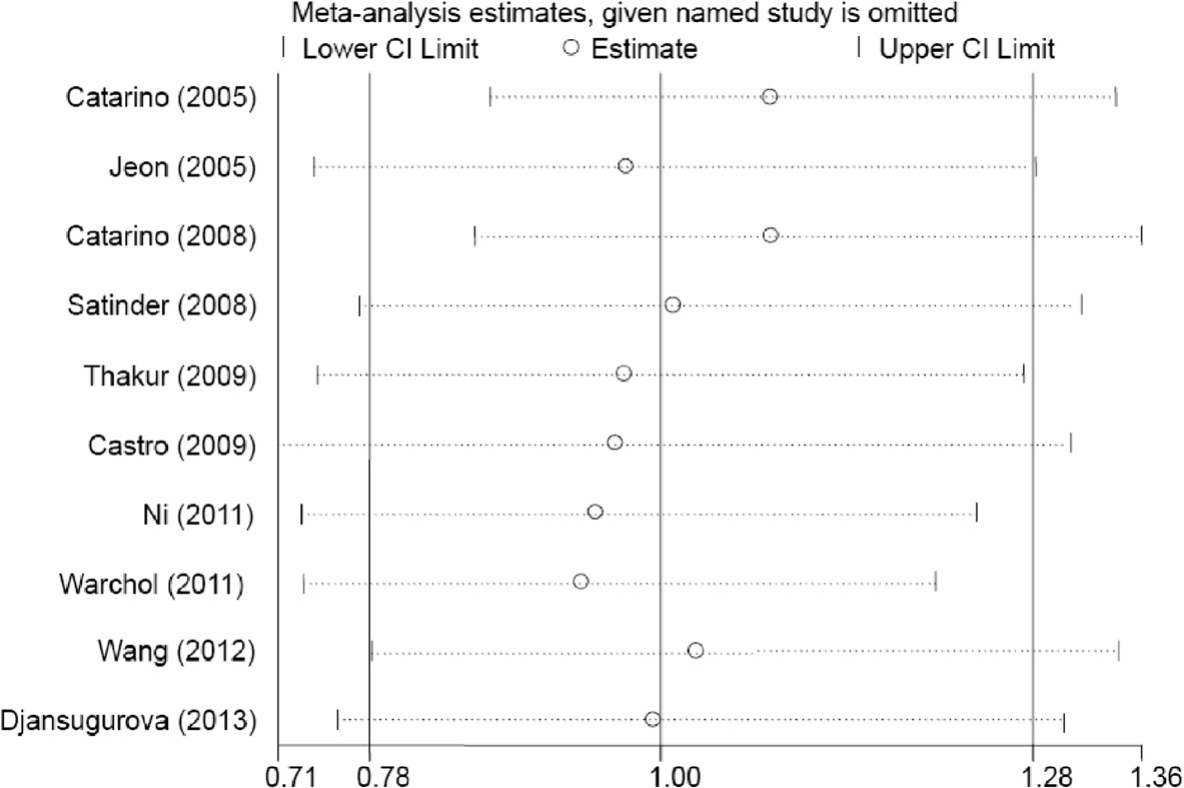
**Sensitivity analysis through deleting each study to reflect the influence of the individual dataset to the pooled ORs in GA + AA model.**

**Figure 4:**
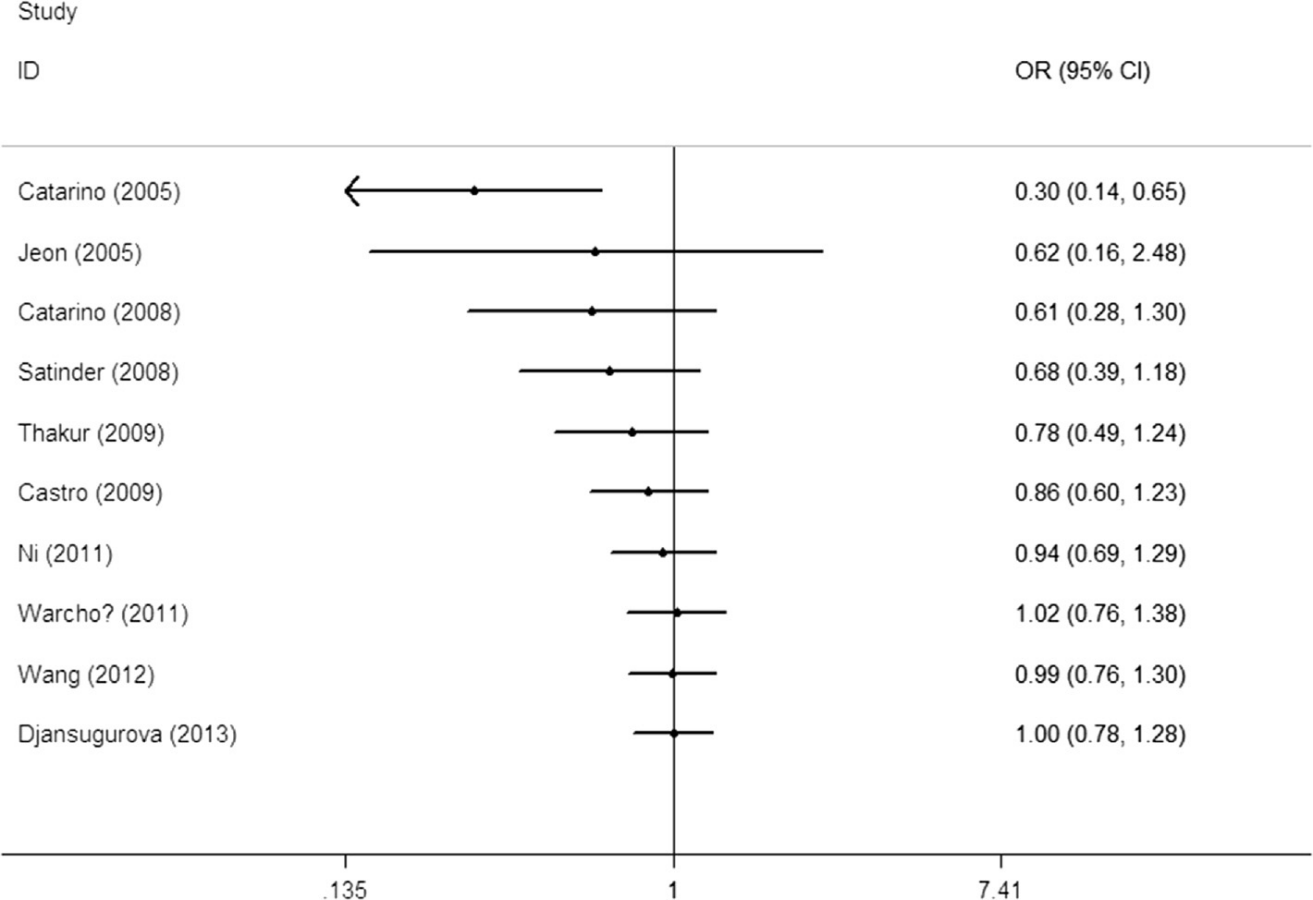
**Cumulative meta-analyses according to publication year in GA + AA vs. GG model.**

Studies included in the meta-analysis were deleted one by one to reflect the influence of an individual dataset on the pooled ORs. The results were consistent (Figure 3 for the dominant model) for all of the researched genetic models, indicating that our results were statistically robust. In the cumulative meta-analysis, the result became negative form the second study of Jeon et al. [12] (Figure 4).

### Publication bias

**Figure 5:**
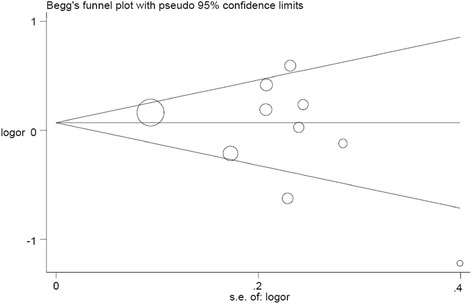
**Funnel plot analysis to detect publication bias for GA + AA model.** Each point represents a separate study.

Funnel plot and Egger’s test were used to estimate publication bias. The shapes of the funnel plots for all genetic models did not reveal any asymmetrical evidence. Figure 5 shows the shapes of the funnel plots of the dominant model used to examine all publications in the meta-analysis. The result was further supported by the analysis of the data with Egger’s test. No significant publication bias was found in this meta-analysis (*P* = 0.643 for A vs. G; GA vs. GG: *P* = 0.427; AA vs. GG: *P* = 0.558; (GA + AA) vs. GG: *P* = 0.423; AA vs. (GG + GA): *P* = 0.884).

## Discussion

Cervical cancer is one of the most dangerous causes of health deficiency and death worldwide. Epidemiological studies have revealed that HPV infection is an important factor contributing to cervical cancer. Furthermore, gene mutation and abnormal tumor cell proliferation may serve critical functions in cancer development.

The CCND1 gene is located at chromosome 11q13 and encodes a key cell cycle regulatory protein with 295 amino acids. CCND1 is an activator of CDK, which can regulate cell division by accelerating/decelerating the transition from G1 to S phase. Over-expression of CCND1 could result in the aberrant proliferation of DNA damage and the accumulation of genetic errors. Some SNPs of CCND1 have been reported. The G-to-A mutation is found in a well-known locus in the boundary of exon 4 and intron 4. This mutation does not alter any amino acid in the protein sequence. CCND1 G870A mutation results in an alternatively spliced transcript with a longer half-life than the CCND1 G allele and thus enables abnormal cells to pass through the G1-S checkpoint easily. Previous studies have demonstrated that CCND1 G870A polymorphism is significantly associated with the development of various cancers, such as breast cancer, prostate cancer, colorectal cancer, and other cancer types [21-25].

In 2005, Catarino et al. firstly find that CCND1 GG polymorphism is associated with a 3.45-fold higher risk for the development of cervical cancer in a Portuguese population (OR = 3.45, 95% CI: 1.47-7.56) [10]. The same results were reported in a subsequent study in 2008 [13]. Similarly, the report by Wang et al. also demonstrated that the GG/GA genotype increased the cervical cancer risk in a Chinese population (OR = 3.31, 95% CI: 1.28-8.59) [19]. By contrast, Satinder et al. found that the AA genotype elevated cervical cancer susceptibility in Indians (OR = 3.7, 95% CI: 1.56-8.87) [14]. Thakur et al. also indicated in 2009 that Indian individuals carrying the AA genotype have a 2.49-fold increased risk of developing cervical cancer (OR = 2.49, 95% CI: 1.51-4.09) [15]. Moreover, other studies by Castro et al. [16] and Warchol et al. [18] found a significant association between the A allele and cervical cancer risk. However, some other studies did not reveal any significant associations between CCND1 G870A polymorphism and cervical cancer.

In 2011, Ni et al. reported the first meta-analysis on the association between CCND1 G870A polymorphism and cervical cancer risk [17]. Only five studies were included in their review. The small sample size without stratified and cumulative analysis may have influenced the strength of their results. Furthermore, in the subsequent meta-analysis of Wu et al., the strength of the conclusion was not enough due to the lack of detailed analysis [11]. Our updated meta-analysis, which includes 10 case–control studies with 2,864 patients and 3,898 controls, did not reveal any significant associations for all genetic models even when stratified analysis was conducted according to ethnicity, study design, and genotyping type. Moreover, the interaction between G870A polymorphism, HPV infectious status and cervical cancer risk was not conducted due to the deficiency of the data. Nevertheless, some current studies have shown that the presence of HPV infection combined with CCND1 G870A polymorphism might increase the risk of cervical cancer, which was consistent with the published reports with positive expression of HPV infection in cervical cancer [19,26,27]. Large sample size with more carefully stratified analyses, more exact statistic techniques and cumulative analysis and meta-regression analysis indicated that our results were statistically robust.

Our analysis has some limitations. First, some heterogeneity still exists despite stratified analysis, and the meta-regression could not be explained successfully. Second, some environmental factors, such as smoking, drinking, and HPV infection, were not included in our meta-analysis because of data deficiency. Third, the sample size was relatively small, which might have yielded false results and inaccurate conclusions.

## Conclusion

In conclusion, this meta-analysis indicated that CCND1 G870A polymorphism may not be a risk factor of cervical cancer development. Large and well-designed case–control studies are needed to validate our findings further.

## Consent

Written informed consent was obtained from the patient for the publication of this report and any accompanying images.

## Abbreviations

HWE: Hardy–Weinberg equilibrium
CCND1: Cyclin D1
SNP: Single nucleotide polymorphism
CNKI: China National Knowledge Infrastructure
OR: Odds ratio
CI: Confidence interval

## References

[1] Jemal A, Bray F, Center MM, Ferlay J, Ward E, Forman D (2011). Global cancer statistics. CA Cancer J Clin.

[2] Garcia-Espinosa B, Nieto-Bona MP, Rueda S, Silva-Sanchez LF, Piernas-Morales MC, Carro-Campos P, Cortes-Lambea L, Moro-Rodriguez E (2009). Genotype distribution of cervical human papillomavirus DNA in women with cervical lesions in bioko, equatorial guinea. Diagn Pathol.

[3] Tang SY, Li L, Li YL, Liu AY, Yu MJ, Wan YP (2014). Distribution and location of daxx in cervical epithelial cells with high risk human papillomavirus positive. Diagn Pathol.

[4] Galderisi U, Jori FP, Giordano A (2003). Cell cycle regulation and neural differentiation. Oncogene.

[5] Sherr CJ (1996). Cancer cell cycles. Science.

[6] Zhang B, Liu W, Li L, Lu J, Liu M, Sun Y, Jin D (2013). Kai1/cd82 and cyclin d1 as biomarkers of invasion, metastasis and prognosis of laryngeal squamous cell carcinoma. Int J Clinical Exp Pathol.

[7] Myong NH (2008). Cyclin d1 overexpression, p16 loss, and prb inactivation play a key role in pulmonary carcinogenesis and have a prognostic implication for the long-term survival in non-small cell lung carcinoma patients. Cancer Res Treat.

[8] Abramson VG, Troxel AB, Feldman M, Mies C, Wang Y, Sherman L, McNally S, Diehl A, Demichele A (2010). Cyclin d1b in human breast carcinoma and coexpression with cyclin d1a is associated with poor outcome. Anticancer Res.

[9] Betticher DC, Thatcher N, Altermatt HJ, Hoban P, Ryder WD, Heighway J (1995). Alternate splicing produces a novel cyclin d1 transcript. Oncogene.

[10] Catarino R, Matos A, Pinto D, Pereira D, Craveiro R, Vasconcelos A, Lopes C, Medeiros R (2005). Increased risk of cervical cancer associated with cyclin d1 gene a870g polymorphism. Cancer Genet Cytogenet.

[11] Wu Y, Fu H, Zhang H, Huang H, Chen M, Zhang L, Yang H, Qin D (2014). Cyclin d1 (ccnd1) g870a polymorphisms and cervical cancer susceptibility: a meta-analysis based on ten case–control studies. Tumour Biol.

[12] Jeon YT, Kim JW, Song JH, Park NH, Song YS, Kang SB, Lee HP (2005). Cyclin d1 g870a polymorphism and squamous cell carcinoma of the uterine cervix in korean women. Cancer Lett.

[13] Catarino R, Pereira D, Breda E, Coelho A, Matos A, Lopes C, Medeiros R (2008). Oncogenic virus-associated neoplasia: a role for cyclin d1 genotypes influencing the age of onset of disease?. Biochem Biophys Res Commun.

[14] Satinder K, Chander SR, Pushpinder K, Indu G, Veena J (2008). Cyclin d1 (g870a) polymorphism and risk of cervix cancer: a case control study in north indian population. Mol Cell Biochem.

[15] Thakur N, Hussain S, Kohaar I, Tabassum R, Nasare V, Tiwari P, Batra S, Bhambhani S, Das BC, Basir SF, Bharadwaj D, Bharadwaj M (2009). Genetic variant of ccnd1: association with hpv-mediated cervical cancer in indian population. Biomarkers.

[16] Castro FA, Haimila K, Sareneva I, Schmitt M, Lorenzo J, Kunkel N, Kumar R, Forsti A, Kjellberg L, Hallmans G, Lehtinen M, Hemminki K, Pawlita M (2009). Association of hla-drb1, interleukin-6 and cyclin d1 polymorphisms with cervical cancer in the swedish population–a candidate gene approach. Int J Cancer.

[17] Ni J, Wang M, Fu S, Zhou D, Zhang Z, Han S (2011). Ccnd1 g870a polymorphism and cervical cancer risk: a case–control study and meta-analysis. J Cancer Res Clin Oncol.

[18] Warchol T, Kruszyna L, Lianeri M, Roszak A, Jagodzinski PP (2011). Distribution of ccnd1 a870g polymorphism in patients with advanced uterine cervical carcinoma. Pathol Oncol Res.

[19] Wang N, Qian X, Wang S, Gao H, Wang L, Huo Y, Zhang S (2012). Ccnd1 rs9344 polymorphisms are associated with the genetic susceptibility to cervical cancer in chinese population. Mol Carcinog.

[20] Djansugurova LB, Perfilyeva AV, Zhunusova GS, Djantaeva KB, Iksan OA, Khussainova EM (2013). The determination of genetic markers of age-related cancer pathologies in populations from kazakhstan. Front Genet.

[21] Comstock CE, Augello MA, Benito RP, Karch J, Tran TH, Utama FE, Tindall EA, Wang Y, Burd CJ, Groh EM, Hoang HN, Giles GG, Severi G, Hayes VM, Henderson BE, Le Marchand L, Kolonel LN, Haiman CA, Baffa R, Gomella LG, Knudsen ES, Rui H, Henshall SM, Sutherland RL, Knudsen KE (2009). Cyclin d1 splice variants: polymorphism, risk, and isoform-specific regulation in prostate cancer. Clin Cancer Res.

[22] Yaylim-Eraltan I, Arikan S, Yildiz Y, Cacina C, Ergen HA, Tuna G, Gormus U, Zeybek U, Isbir T (2010). The influence of cyclin d1 a870g polymorphism on colorectal cancer risk and prognosis in a turkish population. Anticancer Res.

[23] Sergentanis TN, Economopoulos KP (2011). Cyclin d1 g870a polymorphism and breast cancer risk: a meta-analysis comprising 9,911 cases and 11,171 controls. Mol Biol Rep.

[24] Tsai MH, Tsai CW, Tsou YA, Hua CH, Hsu CF, Bau DT (2011). Significant association of cyclin d1 single nucleotide polymorphisms with oral cancer in Taiwan. Anticancer Res.

[25] Akkiz H, Bayram S, Bekar A, Akgollu E, Ozdil B (2010). Cyclin d1 g870a polymorphism is associated with an increased risk of hepatocellular carcinoma in the turkish population: case–control study. Cancer Epidemiol.

[26] Shen SN, Wang LF, Jia YF, Hao YQ, Zhang L, Wang H (2013). Upregulation of microrna-224 is associated with aggressive progression and poor prognosis in human cervical cancer. Diagn Pathol.

[27] Habbous S, Pang V, Eng L, Xu W, Kurtz G, Liu FF, Mackay H, Amir E, Liu G (2012). P53 arg72pro polymorphism, hpv status and initiation, progression, and development of cervical cancer: a systematic review and meta-analysis. Clin Cancer Res.

